# Traditional Herbal Formula NPC01 Exerts Antiangiogenic Effects through Inhibiting the PI3K/Akt/mTOR Signaling Pathway in Nasopharyngeal Carcinoma Cells

**DOI:** 10.1155/2018/5291517

**Published:** 2018-02-13

**Authors:** Li Yanwei, Yang Yinli, Zhanyu Pan

**Affiliations:** ^1^Tianjin Medical University Cancer Institute and Hospital, National Clinical Research Center for Cancer, Tianjin, China; ^2^Key Laboratory of Cancer Prevention and Therapy, Tianjin, China; ^3^Tianjin's Clinical Research Center for Cancer, Tianjin, China; ^4^Department of Integrative Oncology, Tianjin Cancer Hospital, Tianjin, China

## Abstract

Antiangiogenic therapy is vital in nasopharyngeal carcinoma (NPC) treatment. NPC01 has already been successfully used in treating patients with NPC in clinical practice and exerted an excellent antiangiogenetic effect. However, the potential molecular mechanism underlying the antitumor effect of NPC01 has not been well explored. The present study demonstrated that NPC01 could significantly inhibit cell proliferation and induce cell apoptosis in a dose-dependent manner in human NPC cell lines. Furthermore, NPC01 exerted antiproliferative and antiangiogenic effects in NPC xenograft mice. Moreover, the study showed that NPC01 could significantly decrease the expression of angiogenesis-associated factors including hypoxia-inducible factor-1*α* and vascular endothelial growth factor. Additionally, the decreased expression of these angiogenesis-associated factors could be due to the inhibition of the phosphoinositide 3-kinase (PI3K)/protein kinase B (Akt)/mammalian target of rapamycin (mTOR) signaling pathway (PI3K/Akt/mTOR). In conclusion, the results proposed that NPC01 could exert its antitumor effect by suppressing the PI3K/Akt/mTOR signaling pathway. Further studies are warranted to elucidate the molecular mechanism.

## 1. Introduction

Nasopharyngeal carcinoma (NPC) is a head and neck malignant epithelial tumor that shows a clear regional and racial prevalence [1, 2]. NPC is endemic in several areas, particularly in Southeast Asia. A total of 60,000 new cases of NPC and 34,000 deaths attributed to NPC in both sexes were reported in China in the year 2015 [3]. The multifactorial etiology, including infection, genetic predisposition, and environment, has been widely reported, but some other factors still remain unknown [4, 5].

Angiogenesis is the formation of new blood vessels. It is a part of growth and healing. It is also important in cancer growth and spread [6]. Emerging evidence has shown that antiangiogenic therapy is an important part of chemotherapy for several types of cancer [7–9]. Antiangiogenesis can be a potential therapeutic target for NPC treatment, as one of the important biologic features of NPC is the abnormal angiogenesis. Despite the little controversy that radiotherapy is the mainstay of primary treatment, chemotherapy can serve dual purposes of potentiating radiotherapy and eradicating subclinical micrometastasis (PMID: 2064265, 17404741, 8640688, and 2334832). Several studies have demonstrated that compounds derived from plants have anti-inflammatory, antiallergic, antiviral, antiangiogenesis, and anticancer properties [6, 10]. Additionally, increasing lines of evidence suggest that traditional Chinese herbs are sources of compounds that may serve as potential therapeutic drugs in NPC [11, 12]. However, a previous study suggested that a single compound could be effective but did not reflect the formula effects. Therefore, NPC01 was evaluated to determine its* in vitro* and* in vivo* therapeutic effects on NPC cells.

NPC01 is modified from Liang-Ge-Sang, an ancient Chinese herbal formula derived from the formula book “He Ji Ju Fang” of the Song Dynasty (AD 960–1279) in China. “He Ji Ju Fang” has been used for treating inflammation in clinical practice, and Liang-Ge-Sang has been especially used to treat fever, constipation, pharyngitis, and rhinitis in China for centuries [13]. Since some pieces of formula similar to NPC01 have a critical modulatory effect on immune system and have already been used for NPC treatment in clinics, the present study investigated the antitumor effect of NPC01 on NPC cells [14, 15]. The study found that NPC01 could inhibit NPC cell growth* in vitro *and* in vivo* and play antiangiogenic effects putatively by suppressing the PI3K/Akt/mTOR signaling pathway.

## 2. Materials and Methods

### 2.1. Reagents

NPC01 contained five species of medicinal plants purchased from Tianjiang Pharmaceutical Co. Ltd. (Jiangyin, Jiangsu, China, one of the six approved manufacturers of Chinese herbal granules in China) [16]. Every herb of NPC01 was cut into pieces and then mixed together in the ratio of Glycyrrhizae Radix Preparata 60 g,* Rheum palmatum* L. 60 g,* Ligusticum chuanxiong* Hort 75 g,* Coptis chinensis* Franch. 75 g, and* Forsythia suspensa* 120 g. The rule of compositions is based on traditional Chinese medicinal theory, and the compatibility of herbs is due to our clinical experience. The rat doses of were converted from human doses (Chinese Pharmacopeia, 2010) based on body surface areas. According to the body surface areas of human and rat, the oral dose of formula in rats was 500 mg/kg (suspended in 1% CMC-Na), which was equal to its clinical dose (80 mg/kg). The dose was a 10-day treatment for an adult with an average body weight of 60 kg. It was extracted with purified water (390 mL) using a reflux for 2 h at 100°C and then filtered using a 25 *μ*m sieve. The extracts were lyophilized into 27.4 g dry powder, which was stored at −80°C. For experiments, 10 mg dry powder, approximately equivalent to 1‰ of daily dose for one adult person, was dissolved in 100 mL RPMI-1640 culture medium (Gibco, China) and filtered through a 0.45 *μ*m syringe filter before use.

### 2.2. Cell Culture

Human immortalized nasopharyngeal epithelial cell line (NP69) and NPC cell lines 5–8F (high metastasis) were purchased from ATCC and cultured in RPMI 1640 (Gibco, USA) with 10% fetal bovine serum (Gibco, USA). The immortalized nasopharyngeal epithelial cell line NP69 was cultured in keratinocyte serum-free medium (Invitrogen) supplemented with 5% FCS, 25 *μ*g/ml bovine pituitary extract, and 0.2 ng/ml recombinant epidermal growth factor, as suggested. 5–8F was cultured in RPMI 1640 medium (GIBCO, USA) containing 15% FCS by the manufacturer. All the cell lines were grown in a humidified incubator at 37°C with 5% CO2. The cells in the logarithmic growth phase were used for the experiment.

### 2.3. Cell Proliferation Assay

A total of 3 × 10^4^ cells/well were seeded into 96-well plates, incubated overnight, and treated with different concentrations of NPC01 (0, 50, 100, and 200 mg/kg). MTT (Sigma–Aldrich, USA) was added and incubated in the dark at 37°C for 2 h. Absorbance was measured at a wavelength of 490 nm.

### 2.4. Flow Cytometric Analysis

The cell lines were treated with different concentrations of NPC01 (0, 50, 100, and 200 mg/kg). After 24 h, the cells were trypsinized (Sigma) and centrifuged at 1000*g*. The pellets were washed twice using phosphate-buffered saline (PBS). Subsequently, the cells were resuspended and labeled using an Annexin V–fluorescein isothiocyanate (FITC)/propidium iodide (PI) cell apoptosis detection kit according to the manufacturer's protocol (BD Biosciences, NJ, USA).

### 2.5. Quantitative Real-Time Polymerase Chain Reaction

Total RNA was extracted from the NPC cell lines 5–8F and an immortalized nasopharyngeal epithelial cell line, NP69, as well as three NPC biopsies and the paired normal tissues by using TRIzol reagent (Invitrogen). After reverse transcription of the total RNA, the first-strand cDNA was then used as template for detection of LATS2 expression by using quantitative real-time PCR (QT-PCR) with the SYBR Green I chemistry (ABI Inc., USA). The PCR protocol included one cycle at 95°C (3 min), followed by 40 cycles of 95°C (15 s) and 55°C (1 min). The primer sequences were as follows:

  Vascular endothelial growth factor (VEGF) sense,
  5′-TGCCCACTGAGGAGTCCAAC-3′
  VEGF antisense, 5′-TGGTTCCCGAAACGCTGAG-3′,
  Glyceraldehyde-3-phosphate dehydrogenase (GAPDH) sense,
  5′-CGGAGTCAACGGATTTGGCC-3′
  GAPDH antisense, 5′-GTGCAGAGATGGCATGGAC-3′.

### 2.6. Western Blotting

Approximately 5 × 10^5^ cells/well were seeded into 6-well plates and treated with NPC01 or vehicle for 24 h. The protein content was measured using the BCA Protein Assay Reagent (Pierce), and 20 *μ*g of each sample was diluted with 1x lysis buffer. The proteins were separated by sodium dodecyl sulfate-polyacrylamide gel electrophoresis on 4.5–15% gradient gels and transferred onto polyvinylidene fluoride (PVDF) membranes. The membranes were incubated with primary antibodies against phosphorylated Akt (rabbit), mTOR (rabbit), and PI3K (rabbit) which were purchased from Cell Signaling Technology (Danvers, MA, USA). HIF-1a (rabbit) and VEGF (rabbit) were obtained from Abcam company (Cambridge, UK), and antibodies for total Akt (rabbit), mTOR (rabbit), and PI3K (rabbit) were purchased from Cell Signaling Technology (Danvers, MA, USA). The treated cells were collected, and total proteins were extracted using lysis buffer (20 mmol/L Tris-HCl, pH 7.5, 150 mmol/L NaCl sodium pyrophosphate, 1 mmol/L *β*-glycerophosphate, 1 mmol/L Na_3_VO_4_, 1 *μ*g/mL leupeptin, and 1 mmol/L PMSF; Cell Signaling Technology, USA). After centrifugation at 14,000*g* for 15 min at 4°C, the supernatant was collected and the protein concentration was detected using the Bicinchoninic Acid Protein Assay Kit (Pierce Biotechnology, Inc., IL, USA) according to the manufacturer's protocol. Equal amounts of protein were separated using 8%–12% sodium dodecyl sulfate-polyacrylamide gel electrophoresis and transferred onto a polyvinylidene difluoride membrane (Millipore, MA, USA). After blocking with 5% nonfat milk in Tris buffered saline with Tween 20 washing buffer, the membrane was incubated with the specific primary antibodies at 4°C overnight. The blots were labeled with peroxidase-conjugated secondary antibodies. The formed immune complex was visualized using an enhanced chemiluminescence kit (Pierce Biotechnology, Inc.) according to the manufacturer's protocol and exposed to an X-ray film.

### 2.7. A Terminal Deoxynucleotidyl Transferase-Mediated dUTP-Biotin Nick End-Labeling Assay

Cell apoptosis in mouse tumor samples was measured using a terminal deoxynucleotidyl transferase-mediated dUTP-biotin nick end-labeling (TUNEL) assay kit (Roche Diagnostics, IN, USA). Brown nuclei were considered apoptotic. The number of apoptotic cells/1000 cells was recorded in each field of view using a microscope (LZ12; Leica Microsystems GmbH, Wetzlar, Germany) at magnification ×200.

### 2.8. Animal Procedures

All animal procedures, including tumor transplantation, tumor volume measurement, and mouse euthanization, were approved by the Institutional Animal Care and Use Committee at Tianjin Cancer Hospital. The male nude mice, aged 6–8 weeks and weighing 18–20 g, were maintained under specific pathogen-free conditions, because 5–8F is high metastasis nasopharyngeal epithelial cell line. To evaluate the in vivo effect, we performed experiment in NPC cells and human nasopharyngeal epithelial cell line NP69 and the results showed the antitumor effect in 5–8F compared with the control NP69 cell, especially for 5–8F cells. The xenograft tumor model was established by subcutaneously injecting 1 × 10^9^ 5–8 F cells. The mice were daily subjected to the intragastric administration of NPC01 at the indicated dose per mouse or the same volume of PBS as the control for 14 consecutive days after the injection. Tumor volume was calculated according to the formula: volume = 1/2 × length × width^2^. These mice were killed after 2 weeks of NPC01 treatment, and the tumors were harvested for pathological observation. The tumor-inhibiting rate was calculated according to the following formula:

(1) Tumor  inhibiting  rate%=1−mean  of  NPC01  treated  tumor  volumemean  of  vehicle  tumor  volume×100%.

### 2.9. Preparation of Tissues and Histology

The right lower lobes were collected and fixed using 4% paraformaldehyde. The specimens were dehydrated and embedded in paraffin. For histological examination, 4 *μ*m sections of embedded tissue were cut on a rotary microtome, placed on glass slides, deparaffinized, and stained with hematoxylin and eosin. The slides were mounted using Canada balsam (Showa Chemical Co. Ltd., Tokyo, Japan). PAS-positive cells in the epithelium and total epithelial cells were counted, and the percentage of positive cells was calculated. For quantitating airspace in the lungs, the sections with the maximum parenchymal cross sections were selected for morphometric analysis using a digitized image tool. The micrographs were obtained using Image Pro-Plus 5.1 software (Media Cybernetics, Inc. MD, USA).

### 2.10. Statistical Analysis

Data were expressed as the mean ± standard deviation, and a statistical analysis was performed using SPSS version 10.0 (SPSS, Inc., IL, USA). A *P* value < 0.05 was considered to indicate a statistically significant difference.

## 3. Results

### 3.1. NPC01 Inhibited the Proliferation of 5–8F and NP69 Cells

**Figure 1:**
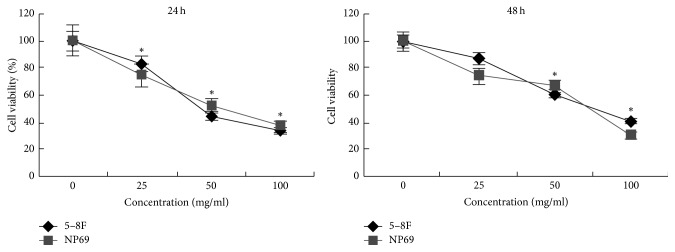
Effect of NPC01 on cell proliferation. 5–8F and NP69 cells were cultured in a 96-well plate and treated with different concentrations of NPC01 for 24 or 48 h. Cell proliferation was assessed using an MTT assay. Values represent the mean ± standard deviation of three independent experiments. ^*∗*^*P* < 0.05.

5–8F and NP69 cells were treated with different concentrations of NPC01 (0, 25, 50, or 100 *μ*g/mL) for 24 or 48 h followed by the MTT assay to investigate the possible antiproliferative effects of NPC01. The result showed that NPC01 could significantly inhibit the proliferation of 5–8F and NP69 cells in a dose-dependent manner (Figure 1).

### 3.2. NPC 01 Promoted NPC Cell Apoptosis

**Figure 2:**
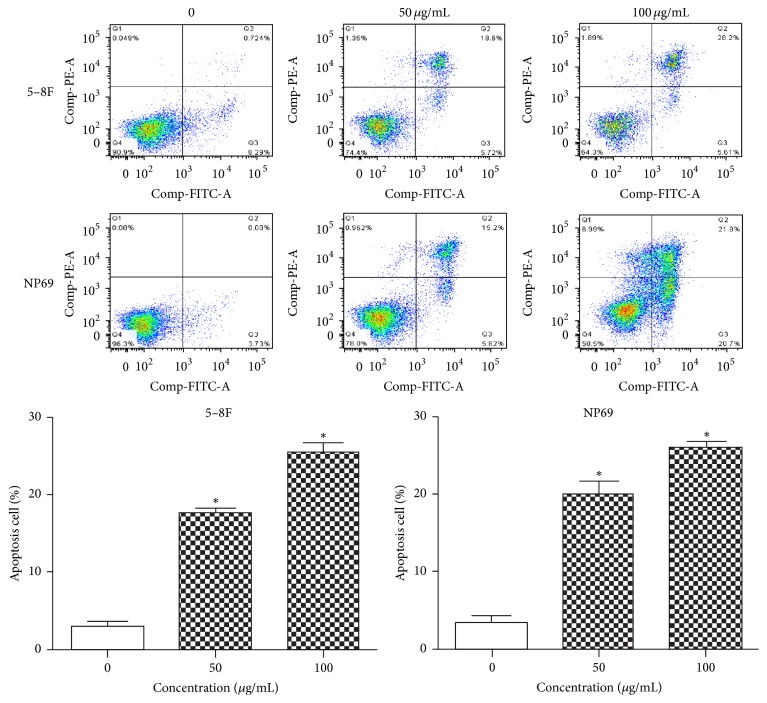
Effect of NPC01 on cell apoptosis. (A) 5–8F and (B) NP69 cells were treated with different concentrations of NPC01 for 24 or 48 h. The cells were collected and labeled with Annexin V–FITC/PI. The signal of Annexin V/PI was analyzed using flow cytometry. Values represent the mean ± standard deviation of three independent experiments. ^*∗*^*P* < 0.05.

5–8F and NP69 cell lines were treated with 25, 50, or 100 *μ*g/mL NPC01 for 24 or 48 h and measured using Annexin V–FITC/PI staining to investigate the possible apoptotic effect of NPC01 on NPC cells. Compared with the control group, NPC cell lines treated with NPC01 showed a significant increase in cell apoptotic population in a dose-dependent manner and 25 *μ*g/mL which has no statistical significance when compared to the control (Data not shown) (Figure 2). These data suggested that NPC01 treatment promoted NPC cancer cell apoptosis.

### 3.3. NPC01 Exerted Significant Therapeutic Activity in NPC Xenografts

**Figure 3:**
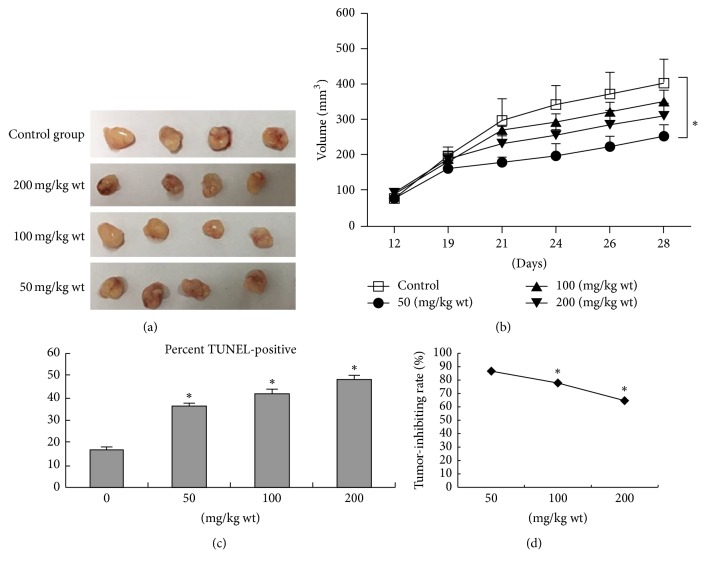
*In vivo* effect of NPC01 in NPC xenograft model. 5–8F cells were subcutaneously injected into the right sides of nude mice for 12 days. Then mice were daily subjected to the intragastric administration of NPC01 at the indicated doses. (a) The tumor masses were photographed at the end of the treatment. (b) The tumor volumes were measured and calculated as described in Materials and Methods. (c) Apoptotic cells were stained using a TUNEL kit and counted as described in Materials and Methods. (d) Groups showed a significant tumor-inhibiting effect. The tumor-inhibiting rate was 87.32%, 77.55%, and 56.16%, respectively. Symbol *∗* indicates *P* < 0.05 compared with the saline-injected mice.

5–8F cells were subcutaneously injected into nude mice to investigate the antitumor effect of NPC01* in vivo*. After 12 days of s.c. 5–8F injection in which the tumor volume was 100–120 mm^3^, the mice were randomized. Tumor volume was calculated using the equation *V* (mm^3^) = 0.5*∗a∗b∗b*. Then NPC xenograft mice were given NPC01 (50, 100, and 200 mg/kg) or saline (negative control) for 2 weeks. At the end of the experiment, autopsies showed that NPC01-treated mice had much smaller tumor masses (Figure 3(a)). The tumor volumes (Figure 3(b)) and average tumor weights significantly decreased in a dose-dependent manner on treating with NPC01 compared with the control group. Additionally, the TUNEL assay also suggested that the number of apoptotic cells in the NPC01 treatment group increased significantly in a dose-dependent manner compared with the control group (Figure 3(c)). After 4-week treatment, the treatment group had a mean tumor volume of 185.9 ± 78.3 mm^3^ versus 402.45 ± 1 : 107.6 mm^3^ for the control group (*P* < 0.05); the tumor-inhibiting rate of high-dose was 56.16% (Figure 3(d)).

### 3.4. Effects of NPC01 on the Expression of VEGF and HIF-1*α In Vivo* and* In Vitro*

**Figure 4:**
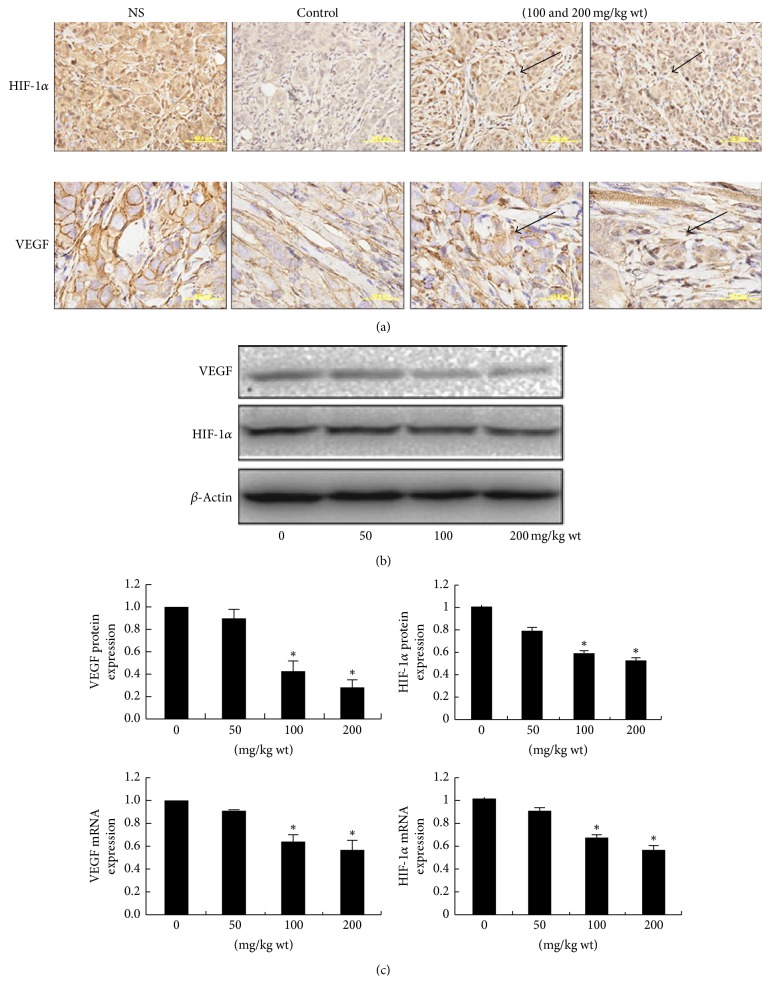
Effect of NPC01 on the* in vivo* and* in vitro* expression level of VEGF and HIF-1*α*. (a) Right lower lobes of mice were dissected and stained with hematoxylin and eosin. (b) 5–8F cells were treated with different concentrations of NPC01 for 24 h. (c) The relative mRNA and protein expression of VEGF were represented as the ratio. Data are shown as mean ± standard error of mean. The symbol *∗* indicates *P* < 0.05 compared with the vehicle group.

The histological sections of xenograft mice with saline and NPC01 treatment were studied to investigate the potential mechanism underlying the antitumor effect of NPC01* in vitro* and* in vivo*. Fewer blood vessels could be found in the NPC01-treated group compared with the saline group. Subsequently, the two representative markers, hypoxia-inducible factor-1*α* (HIF-1*α*) and vascular endothelial growth factor (VEGF), were detected. The results showed that HIF-1*α* and VEGF significantly decreased after NPC01 treatment (Figure 4(a)). Furthermore, the protein and mRNA levels of HIF-1*α* and VEGF were also detected in 5–8F cells treated with NPC01. Parallel with the* in vivo* results, both mRNA and protein levels of HIF-1*α* and VEGF dramatically decreased on NPC01 exposure (Figures 4(b) and 4(c)).

### 3.5. Effect of NPC01 on the PI3K/Akt/mTOR Signaling Pathway

**Figure 5:**
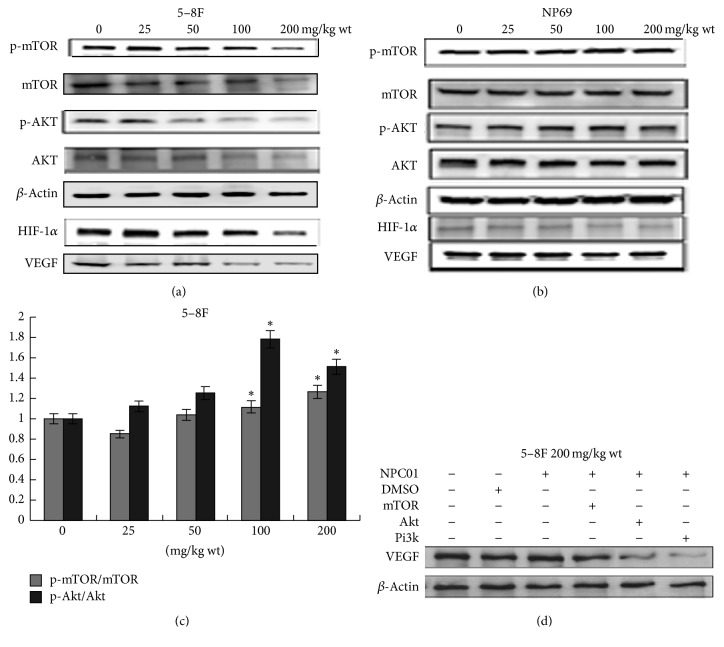
Effect of NPC01 on the PI3K/Akt/mTOR signaling pathway. (a, b) Representative Western blots treated with 25, 50, 100, or 200 *μ*g/mL NPC01 for 24 h or left untreated (control). Protein levels were normalized using *β*-actin as the internal control. (c) Representative blots of the phosphorylated Akt and mTOR, total protein levels of Akt and mTOR, ratio of p-Akt/AKT and p-mTOR/mTOR, and the expression levels in cells. (d) The cells were pretreated with inhibitors of PI3K (LY2940), Akt inhibitor (10 *μ*M), and mTOR inhibitor rapamycin (50 nM) and then stimulated with NPC01 [200 mg/(kg·wt)] for 24 h. Combined results from three independent experiments are shown. ^*∗*^*P* < 0.05, compared with the control.

The PI3K/Akt/mTOR signaling pathway regulates various cellular activities including angiogenesis [17, 18]. 5–8F cells were treated with different concentrations of NPC01 for 24 h to investigate whether the PI3K/Akt/mTOR signaling pathway was involved in the antitumor effect of NPC01. The phosphorylation of Akt and mTOR showed a dose-dependent suppression compared with the vehicle-treated group. A significant inhibitory effect on the PI3K-Akt/mTOR signaling pathway could be detected in the 5–8F cells compared with nasopharyngeal epithelial cell line NP69. Furthermore, the expression levels of HIF-1a and VEGF, which are the downstream targets of the PI3K/Akt/mTOR signaling pathway, decreased dramatically on suppressing the aforementioned pathway (Figures 5(a) and 5(b)). We further analyzed the ratio of protein levels on p-Akt/AKT and p-mTOR/mTOR expression levels in 5–8F cells and found that was significantly and positively correlated with p-PI3K (*P* < 0.01) and p-mTO (*P* < 0.05), and no significance was found in NP69 (Figure 5(c)). 5–8F cells were treated with the inhibitor of PI3K (LY2940), Akt inhibitor (10 *μ*M), or mTOR inhibitor rapamycin (50 nM) to further investigate the involvement of the PI3K/Akt/mTOR signaling pathway in the NPC01-induced antiangiogenic effects. The results demonstrated that the PI3K/Akt inhibitor could reduce the protein expression level of VEGF more effectively compared with the mTOR inhibitor (Figure 5(d)), indicating that NPC01 mainly exerted its antiangiogenic effect by modulating the PI3K/Akt signaling pathway.

## 4. Discussion

Nasopharyngeal carcinoma, as a unique endemic cancer, is the most common head and neck cancer in Southern China. Despite the relatively high response rates to chemotherapy [19, 20] and emerging new drugs, survival in advanced disease cases of NPC is still poor [21, 22]. Hence, it is imperative to explore novel treatments including traditional Chinese medicine (TCM) treatment for patients with NPC. A recent meta-analysis for the clinical efficacy of TCM as a concomitant therapy for nasopharyngeal carcinoma reported significant efficacy of TCM in terms of survival, immediate tumor response, quality of life, immunostimulation, and acute adverse effects [12]. The present study reported that NPC01 could inhibit NPC cell proliferation and induce NPC cell apoptosis in a time- and dose-dependent manner.

Targeting cancer-associated angiogenesis is a promising strategy toward preventing cancer progression or treating cancer [23]. Accumulating evidence established the important role of PI3K/Akt/mTOR signaling pathway in normal and abnormal angiogenesis [17, 18]. Additionally, it has been reported that herbs can impede tumor angiogenesis by suppressing the HIF-1*α*-induced expression of VEGF [24–27]. NPC01 is modified from Liang-Ge-San especially used to treat chronic head and neck diseases in China for centuries [28]. In modern pharmaceutical studies, each herb in NPC01 has different activities, including anti-inflammatory and antiangiogenic [29, 30]. This study demonstrated the important roles of NPC01 in antitumor angiogenesis by inhibiting the PI3K/Akt/mTOR signaling pathway and expression of HIF-1*α* and VEGF both* in vitro* and* in vivo*.

Collectively, the findings indicated that antiangiogenesis could be a promising antitumor strategy for NPC treatment and the PI3K/Akt/mTOR–HIF-1*α*/VEGF signaling pathway axis could be a potential target for antitumor drug screening. NPC01, as a powerful therapeutic TCM formula, can serve as the targeted therapeutic agent for patients with NPC. Further clinical investigation and a detailed understanding of the molecular mechanism underlying the effects of NPC01 would be required for further studies.
